# Editorial: Innovative imaging in neurological disorders: bridging engineering and medicine

**DOI:** 10.3389/fnins.2026.1956446

**Published:** 2026-08-26

**Authors:** Ashraf Elbatanony, Tamer Ahmed Farrag, Mona Zaky, Mohamed Shehata, Mostafa A. Elhosseini

**Affiliations:** 1 Department of Cariology, Faulty of Medicine, Al Azhar University, Cairo, Egypt; 2 Department of Electrical Engineering, College of Engineering, Taif University, Taif, Saudi Arabia; 3 Diagnostic Radiology Department, Faculty of Medicine, Mansoura University, Mansoura, Egypt; 4 Computer Science Program, James C. Bowling School of Business, Midway University, Midway, KY, United States; 5 Computers and Control Systems Engineering Department, Faculty of Engineering, Mansoura University, Mansoura, Egypt; 6 Department of Information Systems, College of Computer Science and Engineering, Taibah University, Yanbu, Saudi Arabia

**Keywords:** artificial intelligence, brain imaging technologies, machine learning, multimodal neuroimaging, neuro-AI integration, neuroimaging, neurological disorders

## 1 Introduction

Neurological diseases such as Alzheimer's Disease, Amyotrophic Lateral Sclerosis (ALS), Autism, Stroke, Brain Tumors, Multiple Sclerosis (MLS), Parkinson's Disease, Epilepsy, Dementia, and Cerebral Aneurysm are very common worldwide. In addition to having always challenged medicine, not only because of its complexity, but also because the brain remains hidden from direct observation. For decades, clinicians depend on signs, symptoms, and limited imaging tools for diagnosis of neurological disorders. Nowadays, the landscape is changing. Thanks to the advances in imaging, from high resolution magnetic resonance imaging (MRI) to functional modalities and enhanced artificial intelligence (AI) analysis, which are transforming how we observe, understand, and moreover early diagnosis of different neurological diseases. This editorial explores how advanced imaging in the rapid evolving era of AI is narrowing the gap between engineering and medicine fields. Researchers are extracting measurable features such as tissue stiffness, microstructure integrity, perfusion pattern, and metabolic signature, that reveal the disease process before symptoms or signs start to appear. This shift is especially important in disorders like Alzheimer's, Parkinson's and MLS, where early intervention can change the disease prognosis.

## 2 Overview of the contributing articles

### 2.1 Neuro-AI integration

By introducing a new AI framework for understanding temporal structure in silent image sequences in CATS (Context-Aware Temporal synthesis) that working without motion cues or audio by using curvature-aware alignment, symmetry-enforced attention and sematic memory. CATS shows achievement up to 15% relative improvement in egocentric video understanding, stable regime separation and accuracy gains on anomalous diffusion dynamic by capturing intrinsic temporal structure rather than dataset-specific cues (Rokaya et al.).

To assess brain activities, they have been using magnetoencephalography (MEG), which is a high temporal and spatial resolution neuroimaging technique. Based on a graph-based long, a short-term memory convolutional neural network (GLCNet) has been used to classify the brain activities to motor imagery (MI) and cognitive imagery (CI) from MEG signals. It has three models for classifying motor and cognitive imagery from MEG signals, a graph convolutional network (GCN), spatial convolution and short-term memory (LSTM) to integrate accurate spatial, temporal and spectral features simultaneously. For best performance evaluation, they used six benchmark models of FBCSP, FBCNet, EEGNet, DeepConvNets, Shallow ConvNet, and MEGNet on two public datasets of MEG-BCI and BCI competition IV dataset. They achieve 78.65% and 65.8% for two classifications and four classifications on the MEG-BCI dataset, respectively (Yang et al.).

Researchers achieved 93.14% accuracy for Alzheimer's disease (AD) early diagnosis. In particular, by using electroencephalogram (EEG) recorded olfactory memory response to rose aroma odors and transformer-based feature fusion to classify AD, mild cognitive impairment (MCI), and healthy subjects. In this study spatial, temporal, and spectral information were extracted from the EEG signal. Signal processing and analysis have been applied, including the common spatial pattern (CSP), in addition to the tangent space matrix covariance together with the tunable Q-factor wavelet transform (TQWT) after integration of multiple feature types (CSP for spatial patterns, covariance for global brain dynamics, and TQWT for time-frequency details). This new approach has accurate capture of EEG signals that helps in early diagnosis of AD, which is more accurate than simple single domain method (Cansiz et al.).

By using modern, fast (high-performance) web framework for building APIs with python 3.7 (FastAPI), and convolutional neural networks (CNNs) in addition to 3,097 brain MRI images and using Grad-CAM for interpretation, investigators found FastAPI deployment enables real-times clinical use for brain tumor diagnosis and classification (Nematzadeh et al.).

### 2.2 Stroke imaging and prognosis

Automated MRI quantification was done in 216 patients with anterior choroidal artery stroke (AChA Stroke) to assess white matter hyperintensity (WMH) and infarct volume to predict 90 days' outcome. The study shows both WMH burden and infarct volume were independently associated with poor functional outcomes (Gao et al.).

Comparing machine learning benchmarking models, atlases, and imaging modalities in a dataset of 238 patients after stroke for predicting severity of aphasia as a language outcome. This study shows best result by combining the Johns Hopkins University Atlas (JHU), lesion location, and the random forest model to identify perisylvian regions as a key predictor (Tilwani et al.).

SynthSR-enhanced MRI, deep learning technology has ability to quantify brain atrophy as an early neurological deterioration (END) in 206 patients presented with anterior choroidal artery (AChA) territory infarction. This study was done by calculation of gray matter fraction (GMF), white matter fraction (WMF), brain parenchymal fraction (BPF), and cerebrospinal fluid fraction (CSPF) to quantify severity of brain atrophy. It shows lower WMF/BPF and higher CSF fraction strong predictor for early neurological deterioration (Gao et al.).

Computed tomography (CT) perfusion for assessment of ischemic core was employed to 91 patients with basilar artery occlusion (BAO) after complete recanalization by thrombectomy in retrospective study. By cerebral blood flow (CBF) threshold assessment, the optimal threshold < 10 ml/100 g/min was observed, and it is a useful method for treatment decisions and follow-up in posterior circulation stroke (Chen et al.).

### 2.3 Neurodegenerative disease biomarkers

Amyotrophic lateral sclerosis (ALS) causes progressive motor affection. Invasive Brain-Computed Interfaces (BCIs; e.g., Neuralink-style microelectrodes), offer communication and control without muscle use. They provide high-resolution signals but carry surgical risk. While, semi-invasive (ECoG, intravascular) has balanced signal quality and safety. On the other hand, non-invasive (EEG, fNIRS) are safer but less accurate (Li et al.).

In a deep learning pipeline study of brain development and structure-function relations in children with unilateral cerebral palsy (uCP), automated measurement of segmenting lesions and quantifying lesion-free brain volume using T1+FLAIR MRI were explored. Results demonstrate that lesion-free volume correlate with motor and visual outcomes, and the thalamic volume has special predictive potentials (Simarro et al.).

T1-relaxation times as ALS early diagnostic marker study by T1-mapping along corticospinal tracts can differentiate ALS patients from control group. ALS patients group shows significant elevation T1 values in corticospinal tracts regions and associated with lower ALS Functional Rating Scale revised (ALSFRS-R) (Dierksen et al.).

A pilot study tested a new rehabilitation approach for patients have post-stroke hemiparesis and unstable gait. Instead of stimulation of brain regions separately, the researchers combined two types of non-invasive brain stimulation at the same time: transcranial direct current stimulation (tDCS) to the supplementary motor area (SMA), which involved rhythm control and postural preparation and transcranial alternating current stimulation (tACS) to the primary motor cortex (M1), which timed precisely to each patient's gait cycle. In this study, a total of 16 stroke patients were randomly assigned to either the real stimulation group or placebo group. Both groups completed 15 treadmill-based gait training sessions over 3 weeks, with peripheral nerve stimulation to assist ankle movement. The real stimulation group showed significant reduced gait variability with better balance scores compared to the control group. Furthermore, improvement in gait variability was strongly correlated with improvement in balance. Therefore, simultaneous stimulation SMA and M1 appears safe, and effective for improving gait stability after stroke (Yamashita et al.).

## 3 Bridging engineering, medicine and future challenges

Despite remarkable progress in advanced neurological imaging, several challenges stand in the routine practice way. As many cutting-edge imaging techniques remain limited to research due to training requirements, or limited availability. In addition, imaging protocol variability, hardware, and interpretation continue to limit large scale clinical applications. Moreover, high-resolution and multimodal imaging generate massive datasets that require sophisticated analysis tools and well-trained personnel. However, AI-driven interpretation holds huge promise, it must be validated, regulated, and merged into existing systems to be trustworthy.

## 4 Conclusions

The merging of engineering innovation and neurological medicine has an important role in reshaping clinical practice. Modern imaging tools are giving clinicians the ability to detect diseases earlier, track progression more precisely and take a decision with greater confidence. These advances are changing how we think about neurological disorders: not as static conditions but as dynamic processes that can be monitored, predicted, and potentially altered. As imaging continues to evolve, it will play a central role in bridging the long-standing divide between what clinicians can observe and what patients' experience. The future of neurology will depend on how effectively we integrate these tools into real world care.

